# Promises and Pitfalls of Whole Exome Sequencing in Therapy-Resistant Chronic Thrombocytopenia in Childhood: A Case Report

**DOI:** 10.3390/jpm16050248

**Published:** 2026-05-02

**Authors:** Eszter Györke, Gábor Benyó, Kristóf Balázs Árvai, Csaba Bödör, László Kereskai, Hajnalka Ábrahám, Barbara Réger, Bálint Egyed, Gábor Ottóffy

**Affiliations:** 1 Department of Pediatrics, Markusovszky Teaching University Hospital, 9700 Szombathely, Hungary; benyo.gabor@semmelweis.hu; 2 Pediatric Center, Semmelweis University, 1085 Budapest, Hungary; egyed.balint@semmelweis.hu; 3 HAS-SU Momentum Molecular Oncohematology Research Group, Department of Pathology and Experimental Cancer Research, Semmelweis University, 1085 Budapest, Hungary; 4 Institute of Pathology, University of Pécs Medical School, 7624 Pecs, Hungary; 5 István Ábrahám Nano-Bioimaging Center, Central Electron Microscopic Laboratory, University of Pécs Medical School, 7624 Pecs, Hungary; 6 Department of Laboratory Medicine, University of Pécs Medical School, 7624 Pecs, Hungary; 7 Department of Pediatrics, University of Pécs Medical School, 7624 Pecs, Hungary

**Keywords:** pediatric hematology, thrombocytopenia, immune thrombocytopenia (ITP), inherited thrombocytopenia, whole exome sequencing (WES), gray platlet syndrome (GPS), personalized therapy

## Abstract

Background: The etiological diagnosis of chronic thrombocytopenia in children remains challenging and is often established by exclusion. In this article, we present the case of a patient in whom we used whole-exome sequencing (WES) to help identify the underlying cause and determine the appropriate treatment. Methods: Whole-exome sequencing was performed to clarify the genetic background of the disease. Based on the results, transmission electron microscopy (TEM) was also carried out to confirm or exclude the pathogenic role of the identified *NBEAL2* gene variant and to assess the presence of gray platelet syndrome. Results: In this patient, despite the presence of the *NBEAL2* gene variant, neither gray platelet syndrome nor a pathogenic role of the variant could be confirmed. However, the genetic findings identified by WES led to numerous additional investigations, causing a considerable burden on both the patient and the family. Conclusions: Our case highlights that WES testing, which is emerging in pediatric hematology practice, offers not only diagnostic advantages but also pitfalls. Whole-exome sequencing has recently emerged as a new diagnostic tool and has been available nationwide in pediatric hematology-oncology care in Hungary for just over two years. While personalized treatment strategies for benign hematologic diseases increasingly rely on high-throughput genetic testing, the clinical application of WES requires a cautious, critical evaluation of results. Despite the method’s promise, the heterogeneity of the findings underscores the need to interpret WES results carefully and to place them in a clinical context in every case.

## 1. Introduction

The underlying cause of newly diagnosed thrombocytopenia at any age is more likely to be an acquired disease than a hereditary disorder [1]. Hereditary thrombocytopenias are rare, heterogeneous disorders that can result in early-onset thrombocytopenia with varying degrees of bleeding. They are characterized by the presence of larger platelets in the blood smear, a positive family history, immunodeficiency, autoimmune symptoms, and splenomegaly [2]. Patients typically respond less well to steroid treatment, immunoglobulin therapy (IVIG), and splenectomy. For this reason, clinical hematologists are forced to perform a wide range of tests to identify the cause of the disease so the patient can receive appropriate therapy. Algorithms and recommendations have changed as the roles of genome and exome sequencing have increased. However, these methods are time-consuming, difficult to access, and extremely expensive, so their use must be decided on an individual basis for each patient, while retaining the traditional methods previously used, such as platelet morphology examination with a light microscope, flow cytometry, and platelet function tests [3].

## 2. Case Report

We detected moderate thrombocytopenia for the first time in a 7-year-old child at age 3.5 years (114 G/L). Her physical status is notable for widespread vitiligo and moderate splenomegaly. During observation, her platelet count was lower (20–50 G/L) on several occasions, but did not require high-dose immunoglobulin (IVIG) treatment. Therefore, following a bone marrow examination, she was treated with oral methylprednisolone (2 mg/kg), which caused her platelet count to rise. The bone marrow examination confirmed hematopoietic activity and ruled out malignancy. During steroid treatment, due to particularly severe thrombocytopenia (5 G/L), the patient was successfully treated with IVIG (1 g/kg) and a temporarily increased dose (4 mg/kg) of steroids (75 G/L). Further tests performed prior to the bone marrow test did not reveal any infection (cytomegalovirus, Epstein–Barr virus, Parvovirus B19, HIV, Toxoplasma, rubella, varicella, herpes simplex virus IgM negative). The autoimmune panel test showed no abnormalities, and immunoglobulin levels were within the normal range for her age (IgGAM). Anisocytosis was observed in the peripheral smear. To clarify the etiology, we initiated several diagnostic tests, including von Willebrand disease, Gaucher disease, hemolytic uremic syndrome, myelodysplasia, systemic lupus erythematosus, liver disease, and Helicobacter pylori infection, all of which were ruled out. Subsequently, due to chronic thrombocytopenia persisting despite the use of azathioprine supplementation, we initiated oral eltrombopag treatment (25 mg/day), which we administered in increasing doses (max. 50 mg/day). We were forced to suspend this treatment due to jaundice, vomiting, and extremely high serum iron levels (147.1 μmol/L), accompanied by an increasing platelet count (61 G/L). We then started subcutaneous romiplostim treatment, which, even at the maximum individualized dose, did not achieve the desired effect (max. 18 G/L). Avatrombopag treatment also proved ineffective. During this period, the child required low-dose steroid treatment (0.5 mg/kg) on several occasions and IVIG therapy (0.8 mg/kg) on four occasions; in all cases, we detected a marked increase in platelet count (80 G/L), but the effect was increasingly short-lived.

Given the progressive nature of the condition and the unclear etiology, we were unable to establish a precise diagnosis for the child based on detailed laboratory and hematological examinations. Since we had tried all the drugs registered for acute and chronic immune thrombocytopenia available in Hungary at the time, as well as combinations thereof, and the child showed a poor response, we performed exome sequencing as part of a personalized examination as the next diagnostic step.

## 3. WES Testing

The WES test was assessed using a virtual gene panel specialized for benign hematological diseases. This method allows us to identify pathogenic gene mutations confirming hereditary thrombocytopenia, which contradicts the diagnosis of chronic ITP due to severe therapy refractoriness and may inform therapeutic decisions in our patient. Furthermore, the increased risk of malignancy in hereditary thrombocytopenia, which is not identified in time, can lead to severe health damage. In many such cases, allogeneic bone marrow stem cell transplantation is a therapeutic alternative. Knowledge of the genetic disease and the specific genetic mutation is also of fundamental importance for the child’s future family planning.

## 4. Method

Next-generation sequencing (NGS) analysis of the entire coding region and splice sites in DNA extracted from peripheral blood samples was executed using the Illumina DNA Prep with Exome 2.5 Enrichment kit on the Illumina NextSeq 2000 instrument (Illumina, San Diego, CA, USA). We used the Dragen Enrichment v4.3.6 (Illumina) application for bioinformatic analysis. The identified nucleic acid sequences were aligned to the GRCh38 human reference genome. We annotated the variants we had found using the Nirvana (Illumina) software (version: v3.23.0). For the pathogenic classification of potential variants, we used the 2015 guidelines of the American College of Medical Genetics and Genomics (ACMG) [4] and the following databases: NCBI-Clinvar, Varsome, and Franklin by Genoox.

## 5. Results

### 5.1. Presumed Pathogenic Variants

The genetic variants identified by the WES can be divided into two groups based on their likelihood of causing disease. The presumed pathogenic variants are shown in Table 1, and variants with unknown clinical relevance are shown in Table 2.

**Table 2:** Variants identified by WES with unknown clinical reference.

Gene	Variant	Variant Identifier	Variant Type	Typical Gene-Related Diseases
*NBEAL2 (NM_015175.3)*	c.806G>A p.(Arg269His) VAF = 100% germline mutation	ClinVar: -	VUS	Gray platelet sy, AR
*VWF (NM_000552.5)*	c.3692A>G p.(Asn1231Ser) VAF = 46.7% germline mutation	ClinVar: 976,752	VUS	von Willebrand disease, type 2A, 2B, 2M, 2N, AR/AD
*PIGT (NM_015937.6)*	c.563C>G p.(Pro188Arg) VAF = 45.1%	ClinVar: -	VUS	Paroxysmal nocturnal hemoglobinuria AD6Smu

Abbreviations: VAF: variant allele frequency, ClinVar: database, VUS: variant of unknown clinical significance. AD: autosomal dominant; AR: autosomal recessive. Explanation: As a result of the identified VWF c.3692A>G p.(Asn1231Ser) sequence change, the amino acid asparagine is replaced by serine in codon 1231 of the protein encoded by the *VWF* gene. As a result of the identified PIGT c.563C>G p.(Pro188Arg) sequence change, the amino acid proline is replaced by arginine in codon 188 of the protein encoded by the *PIGT* gene. As a result of the identified *NBEAL2* c.806G>A p.(Arg269His) sequence change, the arginine amino acid is replaced by histidine in codon 269 of the protein encoded by the *NBEAL2* gene (Figure 1).

**Figure 1:**
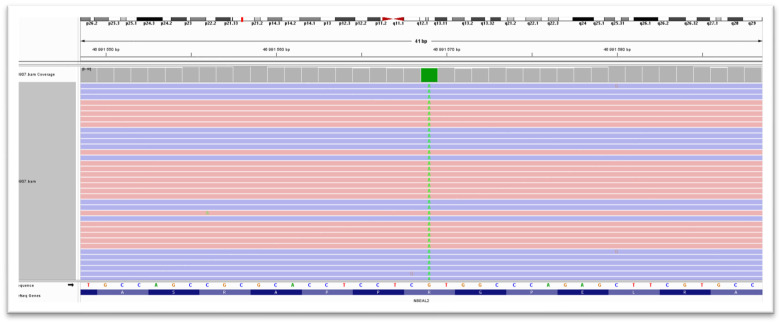
Visualizing the sequence reads and homozygous *NBEAL2* p.(Arg269His) variant in Integrative Genomics Viewer (IGV). Red and blue colors represent strand information. The total coverage of the variants position is 81X (37+, 44−).

### 5.2. Variants with Unknown Clinical Relevance

Following the WES test results, the child has been re-examined for all diseases suggested by the test. In the absence of clinical anemia or hemolysis, paroxysmal nocturnal hemoglobinuria was considered unlikely, and peripheral blood flow cytometry (FCM) did not confirm the disease (negative FCM for CD55 and CD59). Haemostasis testing showed antithrombin III and von Willebrand factor levels within the normal range (antithrombin III activity: 103.6%, von Willebrand factor activity: 64.4%, von Willebrand antigen: 69.7%, VIII clotting factor activity: 135.2%). Platelet function analysis (PFA-200) showed reduced responses to ADP, adenosine diphosphate (ADP), arachidonic acid, and ristocetin (ADP: 38%, arachidonic acid: 41%, ristocetin: 61%; normal range: 70–100%). However, in the setting of thrombocytopenia, prolonged closure time may reflect the low platelet count rather than intrinsic platelet dysfunction. In cases of thrombocytopenia, the PFA-200 cannot reliably distinguish whether the cause of bleeding is a low platelet count or platelet dysfunction [5,6].

**Figure 2:**
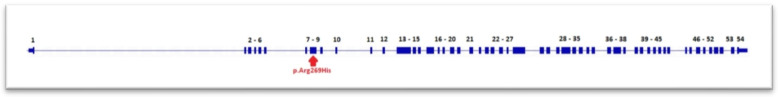
Schematic representation of the *NBEAL2* gene (NM_015175.3), which contains 54 exons, and the homozygous p.(Arg269His) variant located in exon 8.

The most likely disease-causing mutation was a variant in the *NBEAL2* gene (Figure 2), which encodes a BEACH domain-containing protein involved in membrane dynamics and intracellular vesicle transport [7]. Pathogenic variants in *NBEAL2* (neurobeachin-like 2) are known to cause gray platelet syndrome (GPS) [3]. Gray platelet syndrome is a rare inherited disorder characterized by a mild to moderate bleeding tendency, moderate thrombocytopenia, and a significant reduction or absence of platelet alpha granules and the proteins they contain [8]. They possess normal dense (δ) granules, lysosomes, mitochondria, and peroxisomes. Genome-wide association analysis mapped the GPS locus to a 9.4-megabase region on chromosome 3p21 [9,10,11], which contains 197 protein-coding genes, of which 69 have been fully or partially sequenced [11]. Many patients with gray platelet syndrome develop splenomegaly and myelofibrosis [12].

Clinically apparent, inherited thrombocytopenia associated with *NBEAL2* gene mutations is most commonly seen in the form of biallelic (homozygous or compound heterozygous) mutations. Heterozygous carriers, who carry only one mutant allele, are generally not thrombocytopenic, although they may have mild platelet abnormalities. Overall, therefore, the form containing two mutant alleles underlies the classic, clinically pronounced form of GPS (Table 3) [10].

Cases suggestive of both autosomal dominant and autosomal recessive inheritance have been described, suggesting that GPS is likely a genetically heterogeneous disorder with multiple molecular causes. In Japan, Mori et al. Identified 24 affected patients in a single family. In at least one case, transmission from father to son was observed, which is consistent with autosomal dominant inheritance [13].

In a study of 116 individuals with GPS conducted by Gunay-Aygun et al. [10], the majority of patients developed bleeding symptoms in infancy. Thrombocytopenia is usually mild to moderate, but it becomes more severe with age. Myelofibrosis is rare in childhood but tends to appear and worsen in adulthood due to the continuous flow of growth hormones and cytokines into the bone marrow [14]. Thrombocyte aggregation tests showed no abnormalities in the vast majority of cases. In addition to the genetic defect, the presence of large, pale thrombocytes on peripheral blood smear and the absence of alpha granules on electron microscopy were criteria for diagnosis in all cases [10]. All reported cases were homozygous for the abnormalities on chromosome 3, but they considered only patients with a positive transmission electron microscopy (TEM) image as affected. In a 2022 study by Louzil et al., two patients heterozygous for the *NBEAL-2* gene were found to have GPS confirmed during clinical examinations [3]. The variant we examined has not yet been reported in the international literature in individuals with GPS and is not listed in ClinVar. In contrast, they can be found in both heterozygous and homozygous forms in healthy population databases (allele frequency of 0.003% in the GnomAD database, with 1 homozygote—Table 4 and Table 5). 

**Figure 3:**
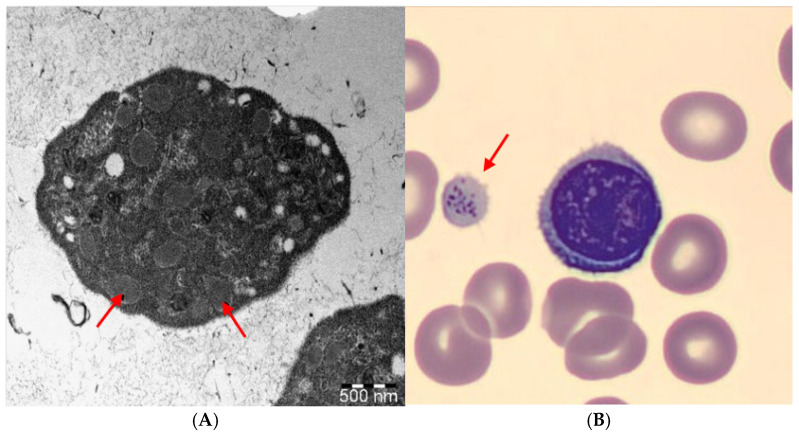
(**A**): TEM image of a platelet showing alpha granules (arrow), whose presence rules out the possibility of GPS. Following isolation, the platelets were fixed with 2.5% glutaraldehyde at 4 °C overnight. After centrifugation in an Eppendorf tube, the sediment was post-fixed with 1% osmium tetroxide at 4 °C for 60 min. The fixed sediment was mixed in 3% agar, followed by cutting 1 mm^3^ blocks and washing them in 0.1 M phosphate buffer for 3 × 10 min. After dehydration with ethanol, they were placed in propylene oxide and embedded in Durcupan resin. The ultrathin sections were placed on copper grids, contrasted with lead citrate and uranyl acetate, and examined using a JEOL JEM 1400Flash transmission electron microscope; Tokio, Japan. (**B**): Image of a large, grayish-colored thrombocyte (arrow) observed in the child’s blood smear with a light microscope.

The variant is a missense mutation that replaces arginine at position 269 of the protein with histidine in exon 8 (out of 54 exons). The variant does not affect the functional protein domain defined by UNIPROT. Pathogenic missense variants are also known in the literature, but gene defects resulting in loss of function are generally identified in patients. Bioinformatic software Due to phenotypic overlap, we performed TEM of the child’s platelets, which did not confirm the presence of GPS (Figure 3). We were unable to conduct a functional test, but given the negative TEM result, we do not currently consider the GPS justified. Based on the WES results and the additional tests performed, we believe that the *NBEAL2* mutation, which previously appeared to play the most significant pathogenic role, is a VUS; it may contribute to the unusual clinical course of the disease, but a definitive diagnosis cannot be established. Based on the article by Yao and Gorevitz published in 2023 and applying the new terminology, we believe that, in our patient’s case, the possibility exists that this is a genetically transitional disease (GTD) [15].

## 6. Discussion

Hereditary platelet disorders (Table 6) are increasingly recognized as an important cause of isolated thrombocytopenia in children, even though they occur at a much lower rate than acquired disorders. This possibility should be considered in patients with “refractory immune thrombocytopenia” if there is a family history of thrombocytopenia or leukemia, or if the platelet count has been low on multiple occasions throughout the child’s life and has occurred in isolation [16,17]. Many of these syndromes are associated with other disorders (immunodeficiency, kidney disease, and risk of malignant tumors). An accurate diagnosis is also important for genetic counseling and to avoid inappropriate interventions (immunosuppressive therapy in cases of presumed refractory ITP). Assessing platelet counts in children and their parents, determining the duration of bleeding symptoms, and identifying related findings in the medical history or physical examination can help distinguish between inherited and acquired causes. Most inherited platelet disorders result from genetic defects in the megakaryocyte lineage, leading to unregulated thrombopoiesis. The number of genetic variants reported to be associated with thrombocytopenia is large and continues to grow [18,19].

**Figure 4:**
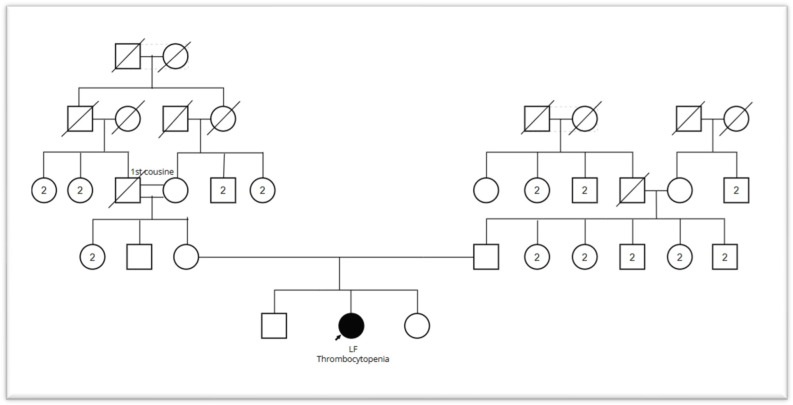
Family tree. Testing for the *NBEAL2* gene mutation was performed only on our patient. We constructed a family tree based on the known occurrence of thrombocytopenia. Every family member had undergone platelet count testing at some point in their lives, and abnormalities were confirmed only in our patient. The sick child’s maternal grandparents are first cousins. Abbreviations in the icons: square2: 2 men, circle2: 2 women; crossed out square: deceased men, crossed out circle: deceased women.

In the treatment of hereditary thrombocytopenia, thrombopoietin receptor agonists (TPO-RA) are expected to be effective, whereas IVIG is not [20]. In our patient’s case, the opposite pattern was observed, contradicting a diagnosis of hereditary thrombocytopenia. However, given the family history of consanguineous marriage (Figure 4), vitiligo involving the entire body, mild splenomegaly, slightly grayer platelets observed on platelet morphology tests, and the increasingly poor response to IVIG, we considered it necessary to perform a WES test. Further testing was necessary due to the *NBEAL2* VUS being homozygous, as it is uncommon for a patient to be homozygous for a phenotype-relevant variant without clinical manifestation.

In our case, we want to highlight the important fact that although the WES tests available today are important, their significance should be assessed. With caution, patients often need to be re-examined (“reverse phenotyped”) based on potentially relevant abnormalities, and the causal role of genetic abnormalities of uncertain clinical significance must be proven. Not all detected gene mutations manifest as clinically apparent diseases. Unfortunately, this cannot always be proven, which can be psychologically stressful for both the family and the treating physicians. The anxiety caused by uncertainty can be reduced through pre-test genetic counseling, which should include informing the family about the possibility of identifying VUSs and that a negative genetic test result does not entirely rule out the possibility of a genetic etiology. All uncertain or inconclusive WES results should be reevaluated every 2–3 years, depending on the patient’s phenotype and clinical course. In this case, it became clear that the findings given to the parents or received on online “findings viewer” sites should only contain clinically relevant information explaining the basic symptoms. It is stressful for both the family and the clinical hematologist who communicates the report, as well as for the healthcare system financially, if the report lists a “multitude” of diseases as possible genetic defects. The question in each case is which genetic abnormality explains the patient’s underlying symptoms. In our opinion, if the complete pathological findings, including all genetic abnormalities, were first analyzed exclusively by the clinical hematologist and geneticist, and then, based on their opinion, the narrowed findings were given to the parents about the diseases that may be relevant as causes, it would be less stressful for the family, the healthcare system, and the clinician. The patient would then need to be clinically re-examined for the specified diseases, and the clinical manifestations of the genetic abnormality confirmed.

Accordingly, after evaluating the case and the series of extended tests performed on the child due to the WES findings, we changed the reporting strategy for WES tests evaluated with our virtual gene panel developed for targeted benign hematological diseases, and we now provide families with much narrower findings.

## Figures and Tables

**Table 1:** Presumed pathogenic variants identified using the ES method.

Gene	Variant	Variant Identifier	Variant Type	Typical Gene-Related Diseases
*F5 (NM_000130.5)*	c.1601G>A p.(Arg534Gln) VAF = 100% germline mutation	ClinVar: 642	Pathogenic	Factor V deficiency activated protein C resistance—thrombophilia, AD
*SERPINC1 (NM_000488.4)*	c.391C>T p.(Leu131Phe) VAF = 42.8% germline mutation	ClinVar: 18,034	Pathogenic	Antithrombin III deficiency—thrombophilia AR/AD

Abbreviations: VAF: variant allele frequency, ClinVar: database, AD: autosomal dominant; AR: autosomal recessive. Explanation: As a result of the identified *F5* c.1601G>A p.(Arg534Gln) sequence change, the amino acid arginine is replaced by glutamine in codon 543 of the protein encoded by the *F5* gene. As a result of the identified *SERPINC1* c.391C>T p.(Leu131Phe) sequence change, the amino acid leucine is replaced by phenylalanine at position 131 of the protein encoded by the *SERPINC1* gene.

**Table 3:** Mutations in the *NBEAL2* gene in patients with clinically manifest GPS, based on the work of Aygun et al. [10].

cDNA	Protein	Exon/Intron	Mutation State
c.2701C>T	p.Arg901X	19	Homozygous
c.881C>G	p.Ser294X	8	Homozygous
c.1163T>C	p.Leu388Pro	11	Homozygous
c.5720+5G>A		Intron 35	Homozygous

**Table 4:** Results of in silico predictions regarding the *NBEAL2* p.(Arg269His) variant. In summary, despite using 11 different algorithms, the effect of the variant on protein function remains uncertain.

Algorithm	Prediction (score)
Revel	Uncertain (0.32)
AlphaMissense	Benign (Moderate) (0.118)
MUT Assesor	Med (2.52)
SIFT	Uncertain (0.054)
MT	Deleterious (1)
FATHMM	Uncertain (−0.6)
DANN	Deleterious (1)
MetaLR	Deleterious (low) (0.55)
PrimateAI	Uncertain (0.67)
BayesDel	Uncertain (−0.17)
GERP	Uncertain (5.27)

**Table 5:** Population frequency of the *NBEAL2* p.(Arg269His) variant in the gnomAD database. In the European population, the variant is considered very rare (0.0026%) with no homozygous occurrence.

Subpopulation	Allele Frequency	Allele Count	Allele Number	Homozygotes
European-Non Finnish (NFE)	0.0026%	31	1,179,830	0
European-Finnish (FIN)	0%	0	53,792	0
Middle Eastern (MID)	0%	0	6082	0
Other (OTH)	0.0144%	9	6239	1
Amish (AMI)	0%	0	908	0
Ashkenazi Jewish (ASJ)	0%	0	29,606	0
African (AFR)	0%	0	74,882	0
East Asian (EAS)	0%	0	44,884	0
South Asian (SAS)	0.0121%	11	91,086	0
American (AMR)	0.0033%	2	60	0

**Table 6:** Classification of inherited thrombocytopenias by platelet size; Abbreviations: MPV: mean platelet volume; MYH9: non-muscle myosin heavy chain [19].

Small Platelets (MPV < 7 fL)	Normal Platelets (MPV 7–11 fL)	Large Platelets (MPV > 11 fL)
Wiskott–Aldrich syndrome	Inherited bone marrow failure syndromes (sy): Fanconi anemia Dyskeratosis congenita Schwachman-Diamond sy Congenital amegakaryocytic thrombocytopenia	Bernard–Soulier sy
X-linked thrombocytopenia	Thrombocytopenia-absent radius (TAR) sy	DiGeorge sy
	Amegakaryocytic thrombocytopenia with radioulnar synostosis	MYH9-related disorders
	Familial platelet disorders with predisposition to myeloid malignancy: Thrombocytopenia 2 (ANKRD26 mutation) Thrombocytopenia 5 (ETV6 mutation)	Paris–Trousseau sy
		Gray platlet sy
		X-linked thrombocytopenia with dyserythropoiesis/thalassemia
		Autosomal dominant deafness with thrombocytopenia (DIAPH1 mutation)
		Sitosterolemia
		ACTN1-related macrothrombocytopenia

## Data Availability

The data presented in this study are available on request from the corresponding authors (the data are not publicly available due to privacy and ethical restrictions, as they relate to a single patient, but may be available upon reasonable request and with appropriate approvals).
